# Usefulness of four-limb blood pressure measurement in prediction of overall and cardiovascular mortality in acute myocardial infarction

**DOI:** 10.7150/ijms.44735

**Published:** 2020-05-23

**Authors:** Po-Chao Hsu, Wen-Hsien Lee, Wei-Chung Tsai, Chun-Yuan Chu, Chee-Siong Lee, Hsueh-Wei Yen, Tsung-Hsien Lin, Wen-Chol Voon, Wen-Ter Lai, Sheng-Hsiung Sheu, Ho-Ming Su

**Affiliations:** 1 Division of Cardiology, Department of Internal Medicine, Kaohsiung Medical University Hospital, Kaohsiung, Taiwan.; 2 Department of Internal Medicine, Kaohsiung Municipal Siaogang Hospital, Kaohsiung, Taiwan.; 3 Faculty of Medicine, College of Medicine, Kaohsiung Medical University, Kaohsiung, Taiwan.

**Keywords:** 4-limb blood pressure, interarm blood pressure difference, interankle blood pressure difference, ankle-brachial index, acute myocardial infarction

## Abstract

Four-limb blood pressure measurement could improve mortality prediction in the elderly. However, there was no study to evaluate whether such measurement was still useful in predicting overall and cardiovascular (CV) mortality in acute myocardial infarction (AMI). Two hundred AMI patients admitted to cardiac care unit were enrolled. The 4-limb blood pressures, inter-limb blood pressure differences, and ankle brachial index (ABI) were measured using an ABI-form device. The median follow-up to mortality was 64 months (25th-75th percentile: 5-174 months). There were 40 and 138 patients documented as CV and overall mortality, respectively. After multivariable adjustment, the ankle diastolic blood pressure (DBP) on the lower side, ABI value, ABI < 0.9, interarm DBP difference, interankle systolic blood pressure (SBP) and DBP differences, interankle SBP difference ≥ 15 mmHg, and interankle DBP difference ≥ 10 mmHg could predict overall mortality (P ≤ 0.025). The ankle DBP on the lower side, interankle DBP difference, and interankle DBP difference ≥ 10 mmHg could predict CV mortality (P ≤ 0.031). In addition, in the Nested Cox model, the model including the ankle DBP on the lower side and the model including interankle DBP difference had the best value for overall and CV mortality prediction, respectively (P ≤ 0.031). In AMI patients, 4-limb blood pressure measurement could generate several useful parameters in predicting overall and CV mortality. Furthermore, ankle DBP on the lower side and interankle DBP difference were the most powerful parameters in prediction of overall and CV mortality, respectively.

## Introduction

Current technology makes simultaneous blood pressure measurement in 4 limbs easy 1, which can provide a complete evaluation of blood pressures and generate a reliable value of blood pressure differences among 4 limbs. An interarm systolic blood pressure (SBP) difference ≥ 10 mmHg was reported to be significantly associated to peripheral vascular disease, coronary artery disease, and increased cardiovascular (CV) and overall mortality 2-5. Besides, an interankle SBP difference ≥15 mmHg was also demonstrated to be correlated to increased CV and overall mortality in patients with hemodialysis 6 and in elderly Chinese population 5.

The ankle-brachial index (ABI) is a simple and noninvasive method to diagnose peripheral artery disease and an ABI < 0.9 has been frequently used to confirm the diagnosis of peripheral artery occlusive disease 7. Additionally, a low ABI was found to be able to predict CV and overall mortality 8-10. In a large national screening database, there is a strong and consistent relationship between ABI level and a history of prevalent myocardial infarction 11. Compared to patients without peripheral artery disease, acute myocardial infarction (AMI) patients with peripheral artery disease were older, had more comorbidities, and had a higher rate of adverse CV events 12.

Sheng et al. found above and beyond arm blood pressure level, the interarm and interankle blood pressure differences and ABI derived from simultaneous 4-limb blood pressure measurement could improve the prediction of mortality in the elderly 5. However, there was no study to evaluate whether simultaneous 4-limb blood pressure measurement was still useful in prediction of CV and overall mortality in AMI patients, a group of patients with high prevalence of peripheral artery disease 13. Hence, the present study was designed to examine the ability of 4-limb blood pressures, inter-limb blood pressure differences, and ABI in prediction of CV and overall mortality in AMI patients. In addition, we also compare the prediction values for CV and overall mortality among these parameters in such patients.

## Materials and Methods

### Study population and design

This observational cohort study consecutively included AMI (ST segment elevation AMI and non-ST segment elevation AMI) patients admitted to our cardiac care unit from November 2003 to September 2004. Patients with atrial fibrillation and limb amputation were excluded. Finally, 200 AMI patients were included in this study. CV and overall mortality data were collected up to December 2018. Mortality data were obtained from the Collaboration Center of Health Information Application (CCHIA), Ministry of Health and Welfare, Executive Yuan, Taiwan.

### Ethics Statement

The study protocol was approved by the institutional review board (IRB) committee of our Hospital. Informed consents have obtained in written form from patients and all clinical investigation was conducted according to the principles expressed in the Declaration of Helsinki.

### Assessment of 4-limb blood pressures, inter-limb blood pressure differences, and ABI

The values of four-limb SBPs, diastolic blood pressures (DBPs), interarm and interankle SBP and DBP differences, and ABI were measured by using an ABI-form device (VP1000; Colin Co. Ltd., Komaki, Japan), which automatically and simultaneously measured blood pressures in both arms and ankles using an oscillometric method 14,15. The ABI-form device measurement was done once in each patient and was performed within 24 hours of admission to cardiac care unit. Interarm blood pressure differences were derived from the SBP and DBP differences between right and left arms. Interankle blood pressure differences were derived from the SBP and DBP differences between right and left ankles. The ABI was calculated by the ratio of the ankle SBP divided by the higher SBP of the arms. After obtaining bilateral ABI values, the lower one was used for later analysis.

### Collection of demographic and medical data

Demographic and medical data including age, gender, body mass index, and comorbid conditions such as diabetes and hypertension were obtained from medical records.

### Definition of overall and CV mortality

All study participants were followed up till December 2018. Survival information and causes of death were obtained from the official death certificate and final confirmation by the Ministry of Health and Welfare. The causes of death were classified by the International Classification of Diseases 10th Revision. Causes of CV mortality were defined deaths due to hypertensive disease, cardiac disease, cerebral vascular disease, ischemic heart disease, myocardial infarction, heart failure, valvular heart disease, and atherosclerotic vascular disease. No participant lost follow-up in our study.

### Statistical analysis

All statistical analyses were performed with SPSS 22.0 software (SPSS, Chicago, IL, USA). Data were expressed as mean ± standard deviation, percentage, or median (25th-75th percentile) for follow-up period. Continuous and categorical variables between groups were compared by independent samples t-test and Chi-square test, respectively. Time to the CV and overall mortality and covariates of risk factors were adjusted using a Cox proportional hazards model. A significant improvement in model prediction was based on the chi-square statistic, which followed a difference in chi-square value and the P value was based on the value compared with the basic model. Kaplan-Meier survival plots were calculated from baseline to time of mortality events. All tests were 2-sided and P values less than 0.05 were considered statistically significant.

## Results

Among the 200 subjects, mean age was 66.2 ± 13.6 years. There were 40 and 160 patients with ST segment elevation AMI and non-ST segment elevation AMI, respectively. The prevalence of interarm SBP difference ≥ 10 mmHg, interarm DBP difference ≥ 10 mmHg, interankle SBP difference ≥ 15 mmHg, and interankle DBP difference ≥ 10mmHg were 14.4%, 5.3%, 33.3%, and 17.7%, respectively.

**Table 1** compares the clinical characteristics between patients with and without mortality. Compared to patients without mortality, patients with mortality were found to have an older age, less male sex, lower BMI, higher prevalence of hypertension, higher left and right arm SBPs, lower left and right ankle DBPs, lower ABI value, higher prevalence of ABI < 0.9, higher interarm DBP difference, higher interankle SBP and DBP differences, and higher prevalence of interankle SBP difference ≥ 15mmHg and interankle DBP difference ≥ 10 mmHg. There were no significant differences for dyslipidemia and smoking (P = 0.834 and P = 0.942, respectively).

The median follow-up to mortality was 64 months (25th-75th percentile: 5-174 months) in all patients. Mortality events were documented during the follow-up period, including CV mortality (n= 40) and overall mortality (n= 138). For in-hospital mortality, there were 8 patients with CV mortality and 30 patients with overall mortality.

**Table 2** shows the predictors of overall and CV mortality using Cox proportional hazards model in the multivariable analysis after adjustment for age, sex, body mass index, diabetes, and arm SBP on the higher side. If we just adjusted above parameters as basic model, age is the only significant predictor for long-term overall and CV mortality (P < 0.001 and P = 0.007, respectively). Then we further added other variables in to the basic model. For blood pressures of lower limb, the ankle DBP on the lower side could predict long-term overall and CV mortality. For ABI data, ABI value itself and ABI < 0.9 could predict overall mortality, but could not predict CV mortality. For interarm blood pressure differences, only DBP difference could predict overall mortality. For interankle blood pressure differences, all of SBP difference, DBP difference, SBP difference ≥ 15mmHg, and DBP difference ≥ 10mmHg could predict overall mortality. However, only DBP difference and DBP difference ≥ 10mmHg could predict CV mortality in our study.

**Figure 1:**
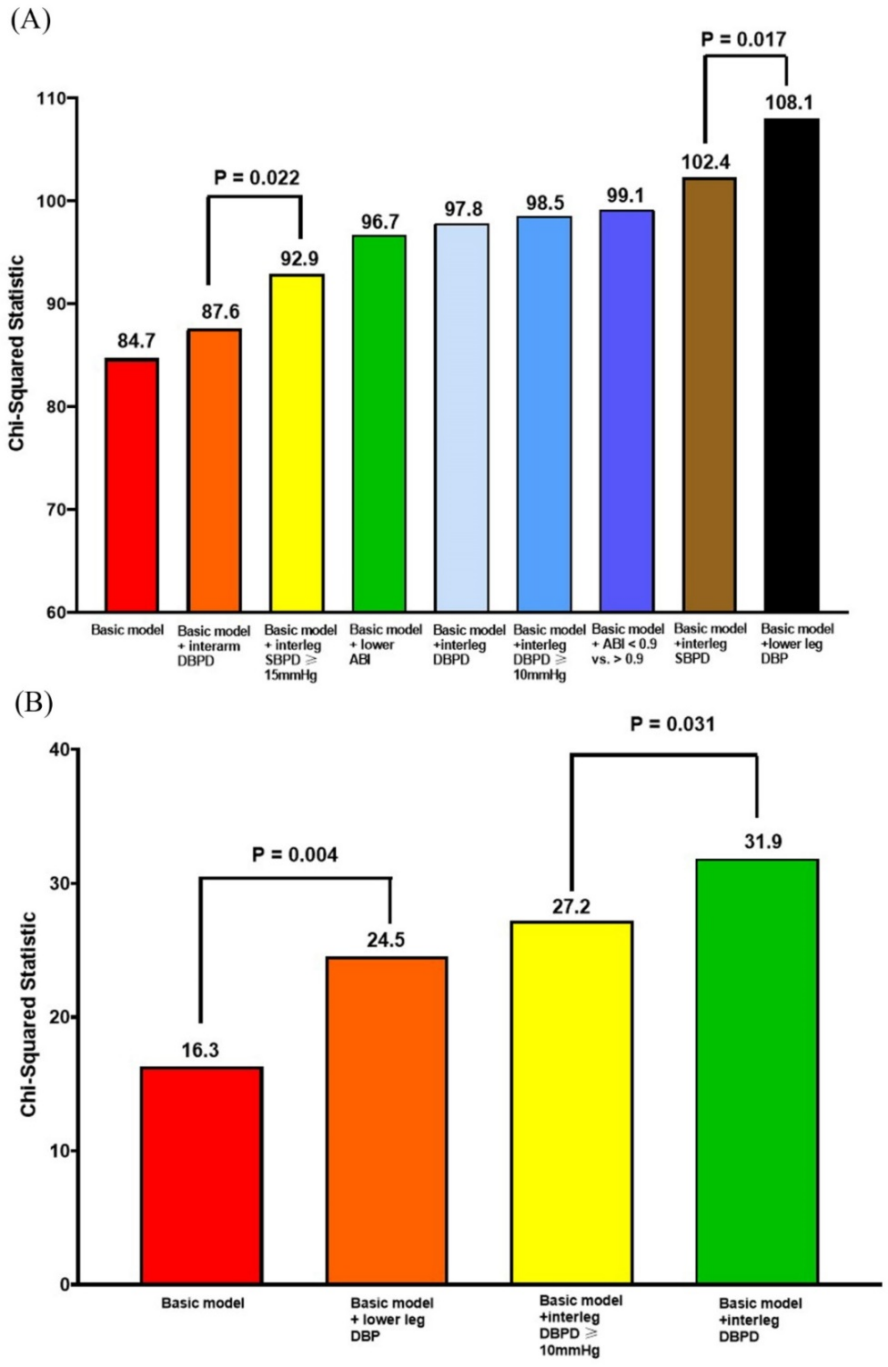
** Nested Cox model for overall mortality and CV mortality.** (**A**) Nested Cox model for overall mortality; (**B**) nested Cox model for CV mortality. Basic model: Adjustment for age, sex, BMI, diabetes, and arm SBP on the higher side. Abbreviations: ABI, ankle-brachial index; BMI: body mass index; CV: cardiovascular; DBP: diastolic blood pressure; DBPD: diastolic blood pressure difference; SBP: systolic blood pressure; SBPD: systolic blood pressure difference.

**Figure 1A** shows the Nested Cox model for overall mortality. The basic model included age, sex, body mass index, diabetes, and arm SBP on the higher side. The basic model could significantly predict overall mortality (Chi-square vale, 84.7, P <0.001). We added the significant parameters in the Table 2, including ankle DBP on the lower side, ABI value, ABI < 0.9, interarm DBP difference, interankle SBP and DBP differences, and interankle SBP difference ≥ 15mmHg and DBP difference ≥ 10mmHg, into the basic model one by one. After comparing the Chi-square values, the model including the ankle DBP on the lower side had the best predictive value for overall mortality. In addition, the model consisting of interarm DBP difference had the lowest prediction value for overall mortality.

**Figure 1B** shows the Nested Cox model for CV mortality. The basic model included age, sex, body mass index, diabetes, and arm SBP on the higher side. The basic model could significantly predict CV mortality (Chi-square vale, 16.3, P = 0.006). We added the significant parameters in the Table 2, including ankle DBP on the lower side, interankle DBP difference, and interankle DBP difference ≥ 10mmHg, into the basic mode one by one. After comparing the Chi-square values, the model including interankle DBP difference had the highest predictive valve for CV mortality.

**Figure 2:**
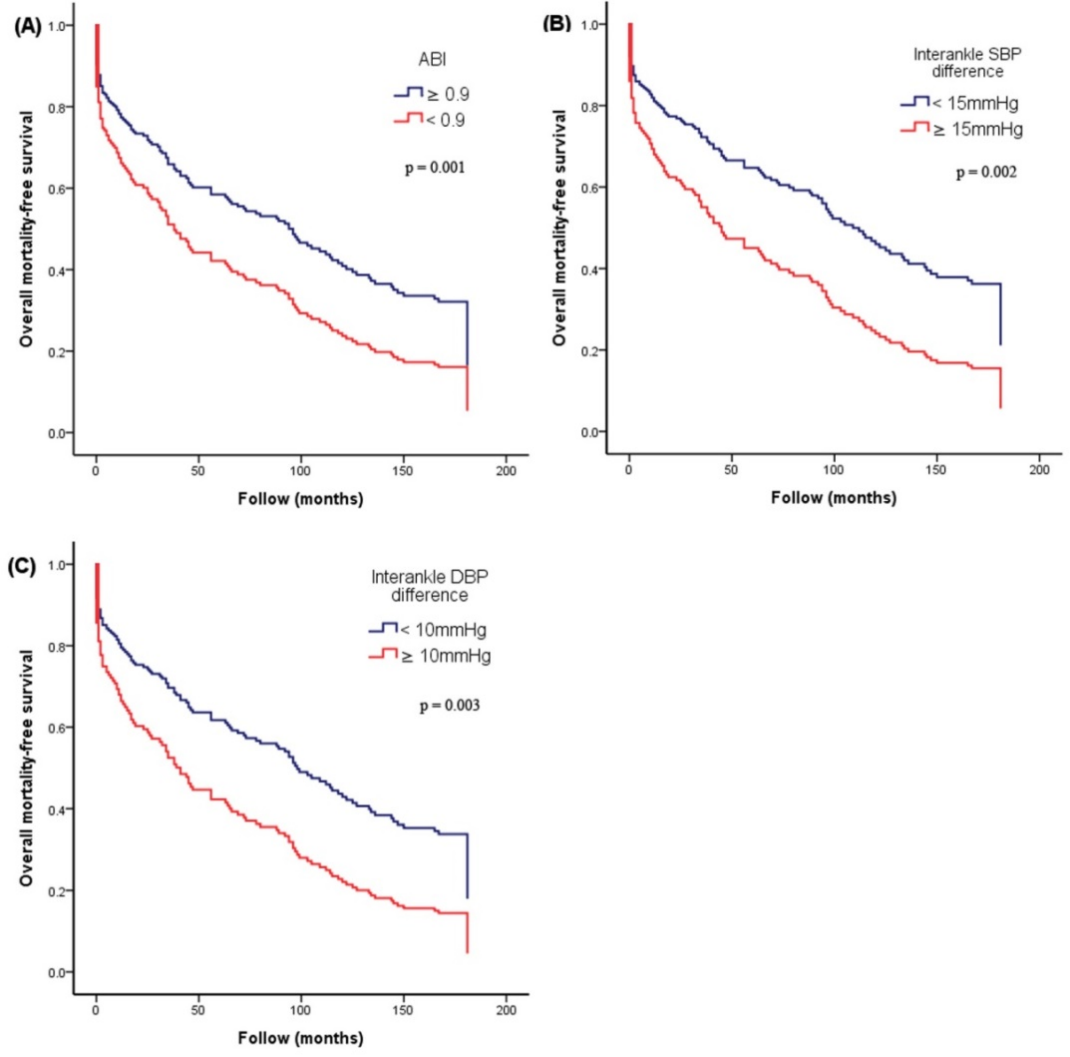
** Kaplan-Meier curves for adjusted overall mortality-free survival.** (**A**) ABI < 0.9 versus ≥ 0.9; (**B**) interankle SBP difference ≥ 15mmHg versus < 15mmHg; (**C**) interankle DBP difference ≥ 10mmHg versus < 10mmHg). Adjustment for age, sex, BMI, diabetes, and arm SBP on the higher side. Abbreviations: ABI, ankle-brachial index; BMI, body mass index; DBP, diastolic blood pressure; SBP, systolic blood pressure.

**Figure 3:**
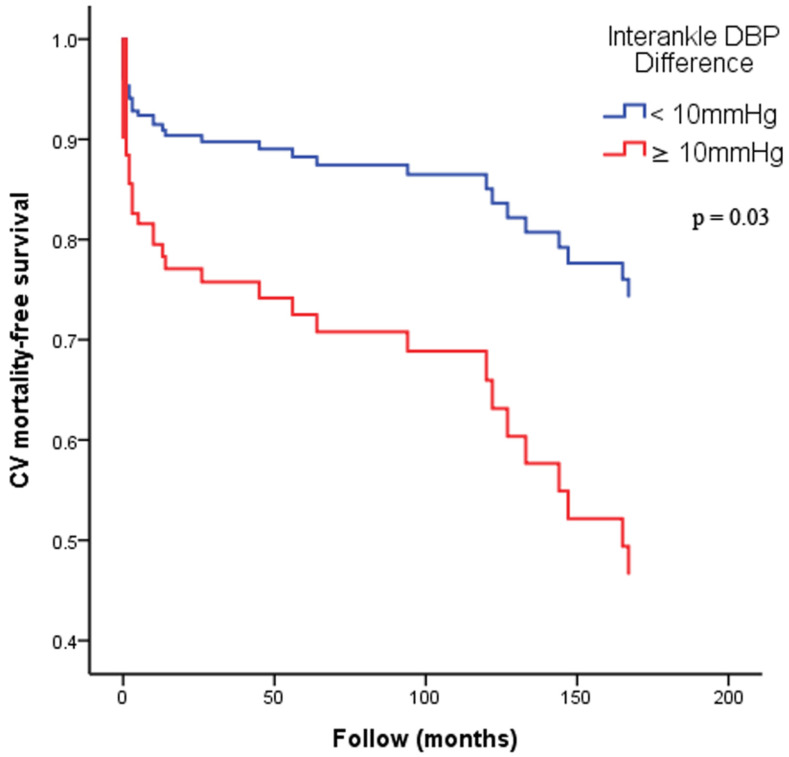
** Kaplan-Meier curves for adjusted CV mortality-free survival.** Adjustment for age, sex, BMI, diabetes, and arm SBP on the higher side. Abbreviations: BMI, body mass index; CV, cardiovascular; DBP, diastolic blood pressure; SBP, systolic blood pressure.

**Figure 2** illustrates the Kaplan-Meier curves for adjusted overall mortality-free survival (**Figure 2A**: ABI < 0.9 versus ≥ 0.9; **Figure 2B**: interankle SBP difference ≥ 15mmHg versus < 15mmHg; **Figure 2C**: interankle DBP difference ≥ 10mmHg versus < 10mmHg). **Figure 3** illustrates the Kaplan-Meier curve for adjusted CV mortality-free survival (interankle DBP difference ≥ 10mmHg versus < 10mmHg).

## Discussion

This study aimed to evaluate the impact of 4-limb blood pressure measurement on the prediction of overall and CV mortality in AMI patients. We found ankle DBP on the lower side, ABI value, ABI < 0.9, interarm DBP difference, interankle SBP and DBP differences, interankle SBP difference ≥ 15 mmHg, and interankle DBP difference ≥ 10 mmHg could predict long term overall mortality. In addition, the ankle DBP on the lower side, interankle DBP difference, and interankle DBP difference ≥ 10 mmHg could predict long term CV mortality. Among these 8 parameters for prediction of overall mortality, 7 parameters could not be obtained without lower limb measurement and 4 parameters belonged to DBP itself or its derived parameters. Among these 3 parameters for prediction of CV mortality, all of them could not be obtained without lower limb blood pressure measurement and all of them belonged to ankle DBP itself or its derived parameters. Hence, for better mortality prediction in AMI patients, simultaneous 4-limb blood pressure measurement was necessary and really had a big impact on the survival prediction. The easily-overlooked ankle DBP and its derived parameters should be taken into consideration.

Simultaneous measurement of blood pressures is preferred in assessment of SBP and DBP differences between limbs because they can avoid overestimation of interarm and interankle blood pressure differences, which may be caused by short-term blood pressure variability or white coat effects 16,17. Therefore, the ABI-form device (VP1000; Colin Co. Ltd., Komaki, Japan) used in this study was a very suitable tool to evaluate the values of interarm and interankle blood pressure difference 14,15.

Interarm blood pressure difference was not an uncommon phenomenon. Typically, interarm SBP difference ≥ 10mmHg was found in 4.4% subjects free of vascular disease. However, the prevalence increased to 7% in diabetic and 13.6% in hypertensive patients 18. In our study, patients with AMI had 14.4% cases with interarm SBP difference ≥ 10mmHg, which was similar with the previous study with hypertensive patients. Interarm SBP difference was associated with CV and overall mortality in various populations, such as patients with hypertension, diabetes, cerebrovascular disease, coronary artery disease, and so on 4,19-23. In a meta-analysis, an interarm SBP difference of ≥ 15 mmHg was associated with a 55% and 68% increase in overall and CV mortality, respectively 22. Not similar with the previous findings, the present study demonstrated that both of the value of interarm SBP difference and interarm SBP difference ≥ 10 mmHg had no association with overall and CV mortality in AMI patients. However, the value of interarm DBP difference could predict overall mortality in the multivariable analysis in our study. DBP was one of the major determinants of coronary perfusion pressure 24,25. Chang et al. ever showed interarm DBP difference ≥ 10 mmHg was related to early neurological deterioration, poor functional outcome, and mortality in stroke patients 26. Hence, interarm DBP difference might be more useful than interarm SBP difference in prediction of overall mortality in AMI patients.

Our previous study showed interankle SBP difference ≥ 15mmHg was independently associated with ABI < 0.9, higher brachial-ankle pulse wave velocity, and increased overall and CV mortality in patients with hemodialysis 6. We also evaluated interankle SBP difference in stage 3-5 chronic kidney disease patients and found interankle SBP difference was associated with rapid progression and progression to renal end points 27. Sheng et al. also found not only increased interarm SBP and DBP differences, but also increased interankle SBP and DBP differences were associated with CV and overall mortality in the Chinese elderly after 4 years follow-up 5. Therefore, interankle blood pressure differences should not be ignored when performing survival analysis. In this study, as shown in **Table 2**, all of four parameters of interankle blood pressure differences and two parameters of interankle DBP differences could predict overall and CV mortality, respectively. Hence, calculation of interankle blood pressure differences, especially the DBP difference, might be very helpful in mortality prediction in patients with AMI.

In our study, in addition to the value of interankle DBP difference and interankle DBP difference ≥ 10mmHg, the ankle DBP on the lower side also could predict overall and CV mortality. Furthermore, in the Nested Cox model, the addition of ankle DBP on the lower side into the basic model had the highest valve in predicting overall mortality. Hence, our results suggested that both of ankle DBP itself and its derived parameters played an important role in overall and CV morality prediction in AMI. Generally speaking, it was not difficult to understand the importance of DBP in patients with AMI. DBP had a J-curve relationship with coronary artery disease and death. Such association was thought to reflect reduced coronary perfusion at low DBP 24,25. Rahman et al. reported that DBP < 60mmHg was associated with increased risk of coronary events and all-cause mortality 24. Protogerou et al. also showed DBP ≤ 60mmHg was associated with reduced survival in the frail elderly 25. These results supported our present finding, i.e. a significant association of DBP with overall and CV mortality. Hence, additional consideration of ankle DBP itself and its derived parameters might provide extra benefit in prediction of overall and CV mortality in AMI patients.

A low ABI was reported to be associated with an increased CV and overall mortality in different population, such as patients with chronic kidney disease 28, hemodialysis 29, diabetes 30, and hypertension 31. In our present study, we consistently found that both of ABI value and ABI < 0.9 also could predict overall and CV mortality in patients with AMI.

Furthermore, age was also shown to be a significant predictor for overall and CV mortality in our study. However, it was not difficult to understand that patients with older age may have higher overall and CV mortality.

### Study limitations

There were some limitations to this study. First, the sample size of our study was not large, but the follow-up period was very long, up to 181 months. Second, we did not adjust hypertension medication in the multivariable analysis because of incomplete data. In our hospital, the chart of patient was not available if he or she did not visit our hospital again more than 10 years. Finally, although simultaneous 4-limb blood pressure measurement was not difficult, many of cardiac or intensive care units lacked an adequate machine to perform such measurement.

## Conclusions

In AMI patients, 4-limb blood pressure measurement could generate several useful parameters in predicting overall and CV mortality. Furthermore, ankle DBP on the lower side and interankle DBP difference were the most powerful parameters in prediction of overall and CV mortality, respectively. Hence, for better mortality prediction in AMI patients, simultaneous 4-limb blood pressure measurement was necessary and really had a big impact on the survival prediction.

## Figures and Tables

**Table 1:** Baseline characteristics of the study population by mortality

Baseline Characteristics	Mortality (+)	Mortality (-)	P value	Total patients
Number	138	62		200
Age (yr)	71 ± 11	55 ± 12	<0.001	66 ± 14
Male gender (%)	65.9%	83.9%	0.011	71.5%
DM (%)	29.7%	25.8%	0.615	28.5%
H/T (%)	47.1%	27.4%	0.013	41.0%
Dyslipidemia (%)	31.2%	30.6%	0.942	31.0%
Smoking (%)	52.6%	50.0%	0.834	51.9%
BMI (kg/m^2^)	23.5 ± 3.9	25.5 ± 3.2	<0.001	24.1 ± 3.8
**Simultaneous 4-limb BP**				
Left arm SBP (mmHg)	126 ± 21	117 ± 17	0.002	123 ± 20
Left arm DBP (mmHg)	71 ± 12	72 ± 12	0.647	71 ± 12
Right arm SBP (mmHg)	128 ± 23	118 ± 18	0.002	125 ± 22
Right arm DBP (mmHg)	72 ± 13	73 ± 13	0.595	72 ± 13
Left ankle SBP (mmHg)	124 ± 37	126 ± 22	0.547	124 ± 33
Left ankle DBP (mmHg)	66 ± 19	73 ± 12	0.003	68 ± 17
Right ankle SBP (mmHg)	126 ± 35	129 ± 23	0.525	127 ± 31
Right ankle DBP (mmHg)	65 ± 19	73 ± 12	0.001	68 ± 18
**ABI data**				
ABI value	0.90 ± 0.23	1.03 ± 0.11	<0.001	0.94 ± 0.21
<0.9 (%)	38.4%	6.5%	<0.001	28.5%
**Interarm BP difference**				
SBP (mmHg)	5.9 ± 5.2	5.0 ± 3.9	0.254	5.6 ± 4.8
DBP (mmHg)	5.1 ± 4.4	3.6 ± 3.0	0.023	4.6 ± 4.0
SBP ≥ 10mmHg (%)	15.9%	11.3%	0.509	14.4%
DBP ≥ 10mmHg (%)	7.2%	1.6%	0.169	5.3%
**Interankle BP difference**				
SBP (mmHg)	17.9 ± 20.0	7.2 ± 6.2	<0.001	14.4 ± 17.3
DBP (mmHg)	8.4 ± 10.0	3.4 ± 2.8	<0.001	6.7 ± 8.7
SBP ≥ 15mmHg (%)	43.3%	12.9%	<0.001	33.3%
DBP ≥ 10mmHg (%)	25.8%	1.6%	<0.001	17.7%

Abbreviations: ABI, ankle-brachial index; BMI, body mass index; BP, blood pressure; DBP, diastolic blood pressure; DM, diabetes mellitus; H/T, hypertension; SBP, systolic blood pressure.

**Table 2:** Predictors of overall and cardiovascular mortality using Cox proportional hazards model

Parameter	Overall mortality		Cardiovascular mortality	
	HR (95% CI)	*P*	HR (95% CI)	*P*
**BP of lower limb**				
Ankle SBP on the higher side	0.998(0.991-1.005)	0.563	1.000(0.987-1.014)	0.954
Ankle DBP on the lower side	0.980(0.969-0.991)	<0.001	0.977(0.957-0.998)	0.031
**ABI data**				
ABI value	0.380(0.162-0.887)	0.025	0.516(0.102-2.603)	0.423
< 0.9	1.917(1.286-2.858)	0.001	2.061(0.982-4.328)	0.056
**Interarm BP difference**				
SBP	1.006(0.970-1.044)	0.746	1.029(0.963-1.100)	0.395
DBP	1.052(1.007-1.100)	0.023	1.045(0.962-1.135)	0.300
SBP ≥ 10mmHg	1.209(0.730-2.002)	0.461	1.957(0.851-4.503)	0.114
DBP ≥ 10mmHg	1.991(0.978-4.056)	0.058	1.591(0.371-6.829)	0.532
**Interankle BP difference**				
SBP	1.019(1.009-1.029)	<0.001	1.011(0.993-1.030)	0.228
DBP	1.030(1.010-1.050)	0.002	1.040(1.010-1.071)	0.009
SBP ≥ 15mmHg	1.809(1.232-2.655)	0.002	0.923(0.444-1.920)	0.831
DBP ≥ 10mmHg	2.087(1.275-3.416)	0.003	2.572(1.093-6.051)	0.030

HR: hazard ratio; CI: confidence interval; other abbreviations as in Table 1. We adjusted age, sex, BMI, DM, and arm SBP on the higher side in a Cox regression model.
